# Next-Generation Sequencing Reveals High Uncommon EGFR Mutations and Tumour Mutation Burden in a Subgroup of Lung Cancer Patients

**DOI:** 10.3389/fonc.2021.621422

**Published:** 2021-04-06

**Authors:** Gang Guo, Gaofeng Li, Yinqiang Liu, Heng Li, Qi Guo, Jun Liu, Xiumei Yang, Tao Shou, Yunfei Shi

**Affiliations:** ^1^Department of Thoracic Surgery, Yunnan Cancer Hospital, Kunming, China; ^2^Department of Thoracic Surgery, First Affiliated Hospital of Kunming Medical University, Kunming, China; ^3^Department of Thoracic Surgery, First People's Hospital of Yunnan Province, Kunming, China; ^4^Department of Medical Oncology, First People's Hospital of Yunnan Province, Kunming, China

**Keywords:** NSCLC, tumour mutation burden, uncommon EGFR mutations, Xuanwei county, NGS

## Abstract

Xuanwei County in Southwest China shows the highest incidence and mortality rate of lung cancer in China. Although studies have reported distinct clinical characteristics of patients from Xuanwei, the molecular features of these patients with non-small cell lung cancer (NSCLC) remain unclear. Here, we comprehensively characterised such cases using next-generation sequencing (NGS). Formalin-fixed, paraffin-embedded tumour samples from 146 patients from Xuanwei with NSCLC were collected for an NGS-based target panel assay; their features were compared with those of reference Chinese and The Cancer Genome Atlas (TCGA) cohorts. Uncommon *EGFR* mutations, defined as mutations other than L858R, exon 19del, exon 20ins, and T790M, were the predominant type of *EGFR* mutations in the Xuanwei cohort. Patients harbouring uncommon *EGFR* mutations were more likely to have a family history of cancer (*p* = 0.048). A higher frequency of *KRAS* mutations and lower frequency of rearrangement alterations were observed in the Xuanwei cohort (*p* < 0.001). Patients from Xuanwei showed a significantly higher tumour mutation burden than the reference Chinese and TCGA cohorts (*p* < 0.001). Our data indicates that patients from Xuanwei with NSCLC harbouring G719X/S768I co-mutations may benefit from treatment with EGFR-tyrosine kinase inhibitors. Our comprehensive molecular profiling revealed unique genomic features of patients from Xuanwei with NSCLC, highlighting the potential for improvement in targeted therapy and immunotherapy.

## Introduction

Lung cancer is the leading cause of cancer-related deaths in China (1). Many patients (57%) are diagnosed with metastatic disease, leading to a 5-year relative survival rate of 5% (2). Approximately 85% of lung cancers are non-small cell lung cancers (NSCLCs), of which lung adenocarcinoma and lung squamous cell carcinoma are the most common subtypes (3). Compared with other regions in China, Xuanwei County in Yunnan Province has the highest mortality rate of lung cancer. The age-standardised mortality rates of lung cancer patients from Xuanwei were six and three times higher than those of patients from rural areas of China, among females and males, respectively (4). Hospitals in Yunnan Province organise free CT examinations to ensure early detection of lung cancer, and many patients are diagnosed at stage I. Xuanwei is rich in smoky (bituminous) coal, which may be associated with the high mortality rate of lung cancer in this area. Retrospective studies have shown that a lifelong use of smoky coal is associated with a 36- and 99-fold increase in mortality in men and women, respectively, compared with smokeless coal use (5, 6). Notably, lung cancer in Xuanwei has some remarkable characteristics, such as higher incidence in non-smoking females, diagnosis at a younger age, rapid tumour progression, multiple lung lesions, poor overall prognosis, and family aggregation (7). Overall, lung cancer patients in Xuanwei may present a distinct subgroup globally, leading researchers to consider whether epidemiological and clinicopathological peculiarities can be interpreted based on genomic features. Recent studies suggested that the NSCLC cohort in Xuanwei harboured a significantly higher co-mutation rate in *EGFR* exons 18 and 20 (8). NSCLCs are often found to have a high tumour mutation burden (TMB), which has been associated with apolipoprotein B mRNA editing enzyme, catalytic polypeptide-like (APOBEC) signatures (9). However, the detailed characteristics of these *EGFR* mutations, comprehensive molecular profiling, and TMB characteristics of patients with NSCLC in Xuanwei are unclear.

We performed comprehensive genomic testing in an NSCLC cohort from Xuanwei. The genomic features of this cohort were compared with those of a reference Chinese NSCLC cohort (1,802 patients, excluding patients from Xuanwei) and data from The Cancer Genome Atlas (TCGA) mainly comprising a Western population from Europe and the US (10).

## Methods

### Patient Enrolment

In total, 1948 Chinese patients diagnosed with NSCLC at the Yunnan Cancer Hospital, First People's Hospital of Yunnan Province, or First Affiliated Hospital of Kunming Medical University were recruited. Formalin-fixed paraffin-embedded tumour samples were collected between December 2017 and January 2019. Matched blood samples were collected as reference controls. Of these patients, 146 were from Xuanwei and defined as the Xuanwei cohort. The remaining 1,802 Chinese patients with NSCLC were defined as the reference Chinese cohort. This study was approved by the Institution Review Board of the First Hospital of Kunming Medical University and conducted according to the Declaration of Helsinki. Informed consent was obtained from all enrolled patients.

### Next-Generation Sequencing (NGS)

All tumour tissues and matched blood samples underwent targeted NGS-based genomic testing (OrigiMed, Shanghai, China) in a College of American Pathologists-accredited and Clinical Laboratory Improvement Amendments-certified laboratory (11). Approximately 50 ng of cancer tissue DNA was extracted from 40 mm formalin-fixed paraffin-embedded tumour samples and blood samples using the DNA Extraction Kit (Qiagen, Hilden, Germany), according to the manufacturer's instructions. All coding exons and selected introns of targeted genes were captured for hybridisation capture panel and then sequenced on an Illumina NextSeq-500 Platform (Illumina Incorporated, San Diego, CA). For formalin-fixed paraffin-embedded samples, sequencing depth was 900 × mean coverage (minimum 700 ×); for matched blood samples, sequencing depth was 300 ×. Genomic alterations, including single nucleotide variants, short and long insertions/deletions, copy number variations, and gene rearrangements, were subjected to advanced analysis. TMB score was calculated from a 450-gene panel data (Supplementary Table 1) for each sample by counting the number of somatic mutations, including coding single nucleotide variants and insertions/deletions, per megabase (Mb) of the sequence examined. Known somatic mutations in the Catalogue of Somatic Mutations in Cancer (COSMIC; https://cancer.sanger.ac.uk/cosmic/signatures) and known germline polymorphisms in the U.S. National Centre for Biotechnology Information's Single Nucleotide Polymorphism Database were not counted (12). Particularly, 35 and 111 tumour samples were subjected to 37 and 450 cancer-related gene panel testing (Supplementary Tables 1, 2), respectively. TMB analysis was available for 111 cases. A high TMB (TMB-H) was defined as ≥10 muts/Mb, and a low TMB (TMB-L) was defined as <10 muts/Mb. Mutational signature analysis was conducted using the deconstructSigs package v1.8.0. All the detected somatic mutations, including synonymous mutations in the cohort, were imported for signature analysis. Finally, the weights of 30 known cancer mutation signatures in COSMIC were generated (13, 14). Uncommon *EGFR* mutations were defined as mutations other than L858R, exon 19del, exon 20ins, and T790M (15).

### Response Evaluation

All nine patients received oral EGFR-tyrosine kinase inhibitor (TKI) treatment. Radiological follow-up was performed first, after 1 month, and then, once every 2 months, via computed tomography of the thorax and upper abdomen. Response was assessed according to the Response Criteria in Solid Tumours (RECIST) 1.1 (16). Progression-free survival was defined as the interval from the date of initiation of EGFR-TKI therapy to the date of disease progression or death from any cause, whichever occurred first.

### Statistical Analyses

Statistical analyses were performed using the R Statistical Software package (R Foundation for Statistical Computing, Vienna, Austria). To analyse differences in continuous variables and TMB, the Wilcoxon test was performed when comparing each two groups, and the Kruskal-Wallis test was performed when comparing all three groups. The Chi-square or Fisher's exact tests was used for association of categorical variables. The threshold for statistical significance was set at *p* < 0.05. The significance associated with each symbol is as follows: ^***^p < 0.001; ^**^p < 0.01; and ^*^p < 0.05.

## Results

### Patients

Clinicopathological data of Xuanwei and reference Chinese patients with NSCLC are summarised in Table 1. The median age of the Xuanwei cohort was lower than that of the reference Chinese cohort (55 vs. 66 years, *p* < 0.001). Patients from Xuanwei showed less incidence of squamous cell carcinoma than reference Chinese patients (7.5 vs. 12.5%, *p* = 0.008). According to the pathology and medical history following the American Journal of Critical Care Cancer Staging Manual, patients were classified based on their main clinical stages (I–IV). The Xuanwei cohort contained more stage I–II patients than the reference Chinese cohort (68.5 vs. 45.7%, *p* = 0.0018). Moreover, Xuanwei cases had a greater cancer-related family history than reference Chinese cases (41.8 vs. 26.6%, *p* < 0.001), with most showing a family history of lung cancer (96.7%).

**Table 1:** Clinicopathological baseline characteristics of patients from Xuanwei and reference Chinese patients with NSCLC.

**Characteristics**	**Xuanwei cohort (*N* = 146)**	**Reference Chinese cohort (*N* = 1,802)**	***p-*value**
Gender [*N* (%)]			0.7914
Male	82 (56.2%)	985 (54.7%)	
Female	64 (43.8%)	817 (45.3%)	
Age (median years, range)	55 (36–78)	66 (22–92)	<0.001
Stage [*N* (%)]			<0.001
I	86 (58.8%)	632 (35.1%)	
II	14 (9.6%)	192 (10.7%)	
III	22 (15.1%)	356 (19.8%)	
IV	22 (15.1%)	620 (34.3%)	
Unknown	2 (1.4%)	2 (0.1%)	
Smoking history [*N* (%)]			0.1899
Yes	58 (39.7%)	550 (30.5%)	
Never	86 (58.9%)	1,046 (58.1%)	
Unknown	2 (1.4%)	206 (11.4%)	
Histology [*N* (%)]			0.008
Adenocarcinoma	131 (89.8%)	1,567 (87.0%)	
Squamous cell carcinoma	11 (7.5%)	225 (12.5%)	
Others	4 (2.7%)	10 (0.5%)	
Family history [*N* (%)]			<0.001
Yes (lung cancer family history)	61 (41.8%) (59, 96.7%)	479 (26.6%) 232 (48.4%)	
No	83 (56.8%)	1183 (65.6%)	
Unknown	2 (1.4%)	140 (7.8%)	
Lesion number [*N* (%)]			
1	49 (33.6%)	–	
≥2	91 (62.3%)	–	
Unknown	6 (4.1%)	–	

### Driver Genes in the Xuanwei NSCLC Cohort

**Figure 1:**
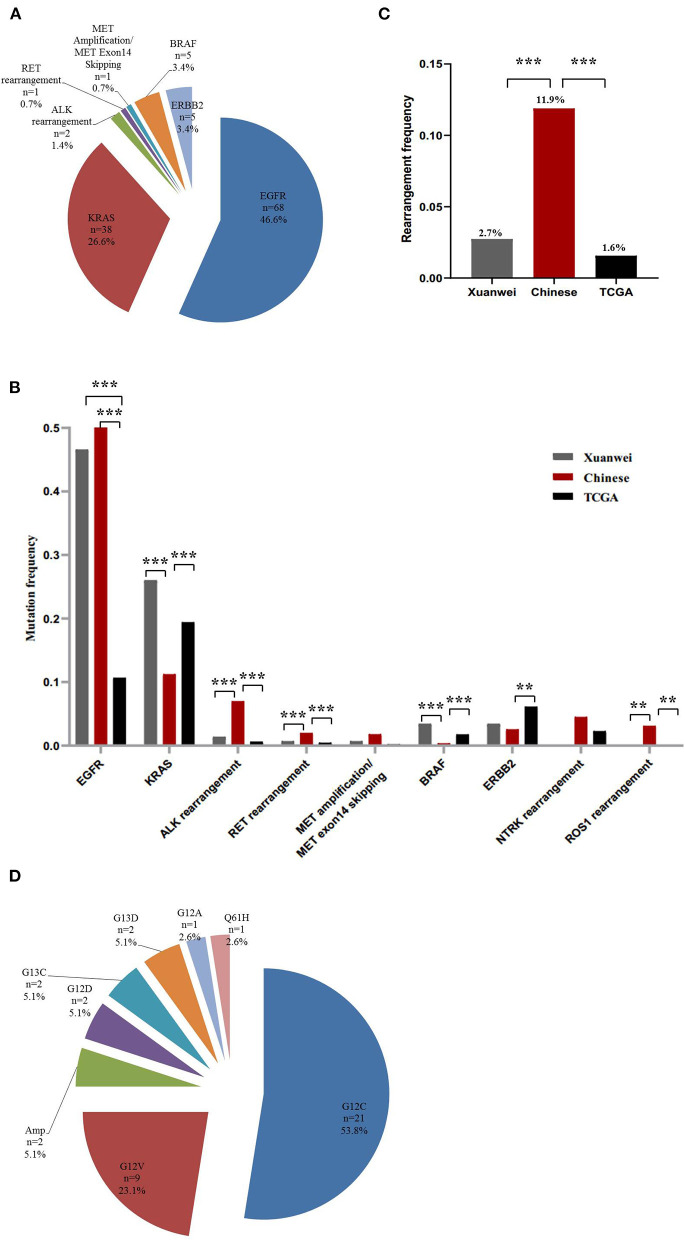
Driver gene mutation profile in the Xuanwei NSCLC cohort. **(A)** Mutation frequency of nine genes in Xuanwei cohorts. **(B)** Composition of the alteration type in the nine genes among the three groups, namely the Xuanwei cohort (left column), reference Chinese cohort (middle column), and TCGA cohort (right column). **(C)** Composition of the rearrangement alterations among the three groups. **(D)** Distribution of *KRAS* mutation subtypes in patients from Xuanwei. ***p < 0.001, **p < 0.01, and *p < 0.05.

The nine driver genes of NSCLC found in patients from Xuanwei are summarised in Figure 1A. Comparison of the mutational profile of driver genes in the three cohorts showed significant differences (Figure 1B). The *KRAS* mutation frequency in the Xuanwei cohort was significantly higher than that in reference Chinese groups (26.3 vs. 11.2%, *p* < 0.001). The frequency of rearrangement alterations identified in the Xuanwei cohort was lower than that in the reference Chinese cohort (2.7 vs. 11.9%, *p* < 0.001). Rearrangement in *ROS1* and *NTRK1/2/3* were not found in the Xuanwei cohort (Figures 1B,C). The most common *KRAS* mutation in the Xuanwei cohort was *KRAS* G12C (53.8%), followed by *KRAS* G12V (23.1%) (Figure 1D).

### *EGFR* Mutation Profile of the Xuanwei Cohort

**Figure 2:**
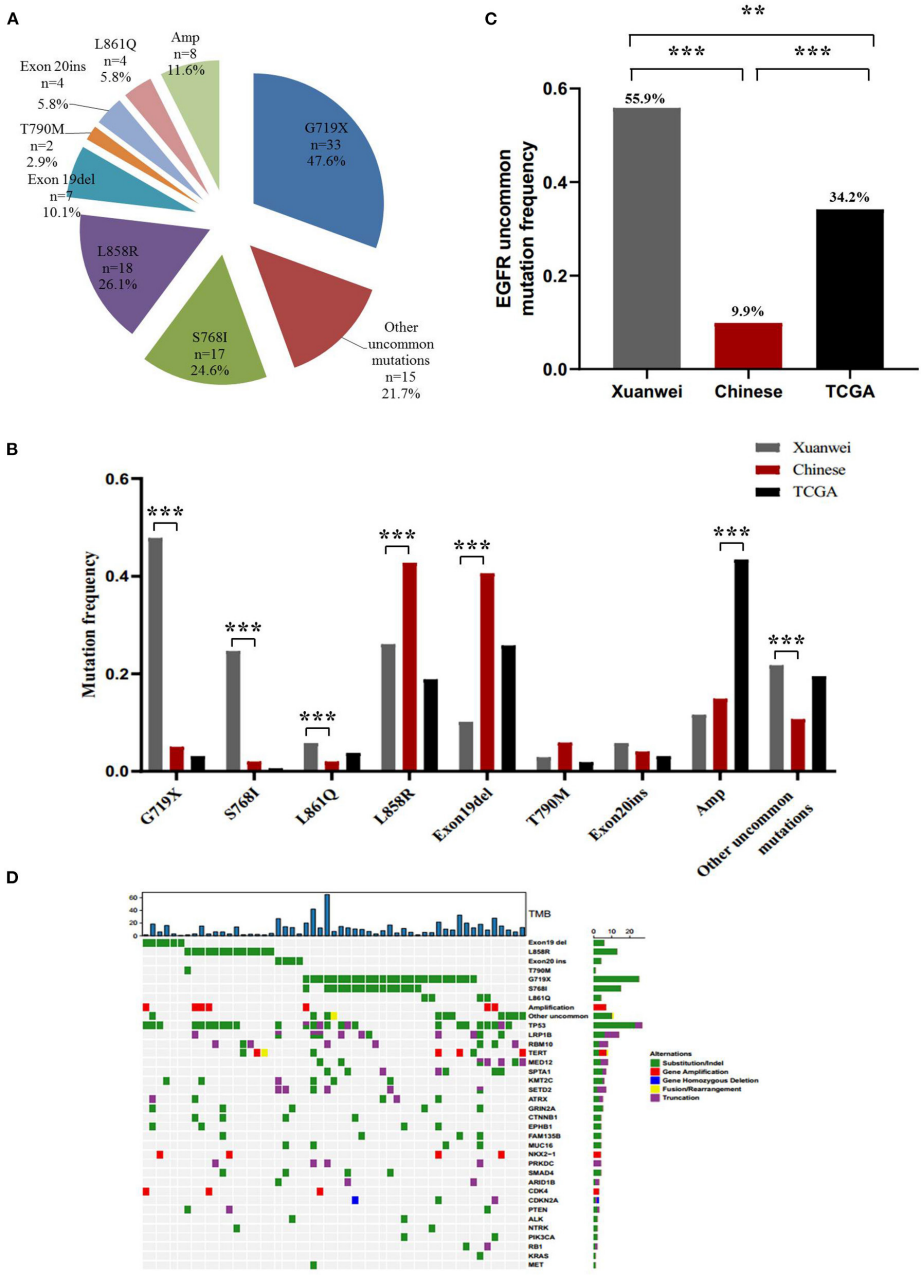
*EGFR* mutation spectrum in the Xuanwei, reference Chinese, and TCGA NSCLC cohorts. **(A)** *EGFR* mutation subtypes of patients from Xuanwei. **(B)** Comparison of *EGFR* mutation profile among the three groups. **(C)** Ratio of uncommon *EGFR* mutations in the three groups. **(D)** Mutation profiles of patients from Xuanwei with *EGFR* genomic alterations. Mutant frequencies in the cohort are shown on the right. TMB for each patient is shown at the top. ***p < 0.001, **p < 0.01, and *p < 0.05.

A higher mutation frequency of *EGFR* was observed in the Xuanwei NSCLC cohort than in TCGA cases (46.6% vs. 10.7%, *p* < 0.001), although this frequency was comparable to that in reference Chinese cases (46.6 vs. 50.3%, *p* = 0.44) (Figures 1A,B). Notably, comparison of *EGFR* mutation subtypes demonstrated that patients from Xuanwei, compared to reference Chinese and Western patients, harboured a striking mutation pattern of higher *EGFR* G719X (47.6% vs. 5.0 vs. 3.1%, *p* < 0.001) and S768I (24.6% vs. 2% vs. 0.6%, *p* < 0.001) mutations, whereas classical *EGFR*-sensitive mutations, such as L858R (26.1% vs. 42.8% vs. 18.9%, *p* < 0.001) and exon 19del (10.1% vs. 40.6% vs. 25.8%, *p* < 0.001), showed significantly lower frequencies (Figures 2A,B; Supplementary Figures 1A,B).

Uncommon *EGFR* mutations were the predominant *EGFR* mutation type in the Xuanwei cohort compared to the reference Chinese cohort (55.9 vs. 9.9%, *p* < 0.001) (Figure 2C). The most commonly co-mutated genes are shown in Figure 2D. Tumours harboured a higher ratio of *EGFR* G719X and S768I co-mutations, which were mutually exclusive of L858R and exon 19del (Figure 2D). The clinicopathological characteristics of the Xuanwei NSCLC cohort with either uncommon or common *EGFR* mutations are summarised in Table 2. Patients with uncommon *EGFR* mutations were more likely to have a family history of cancer than those with common *EGFR* mutations (*p* = 0.048).

**Table 2:** Comparison of characteristics of patients from Xuanwei with NSCLC harbouring common and uncommon *EGFR* mutations.

**Characteristics**	**Overall**	**Common**	**Uncommon**	***p-*value**
	**(*N* = 68)**	**(*N* = 30)**	**(*N* = 38)**	
**Gender**				
Male	29 (42.6%)	12 (40%)	17 (44.7%)	0.81
Female	39 (57.4%)	18 (60%)	21 (55.3%)	
**Age (years)**				
Median age (range)	54 (36–78)	53 (36–72)	55 (38–78)	0.08
**Stage**				
I	44 (64.7%)	19 (63.3%)	25 (65.8%)	0.95
II	4 (5.8%)	2 (6.7%)	2 (5.5%)	
III	8 (11.8%)	4 (13.3%)	4 (10.5%)	
IV	11 (16.2%)	4 (13.3%)	7 (18.2%)	
Unknown	1 (1.5%)	1 (3.4%)	0	
**Histology**				
Adenocarcinoma	67 (98.5%)	29 (96.7%)	38 (100%)	
others	1 (1.5%)	1 (3.3%)	0	
**Family history**				0.048
Yes	28 (41.2%)	8 (26.7%)	20 (52.6%)	
No	39 (57.3%)	21 (70%)	18 (47.4%)	
Unknown	1 (1.5%)	1 (3.3%)	0	
**Smoking history**				
Yes	19 (27.9%)	9 (30%)	10 (26.3%)	0.43
Never	48 (70.5%)	20 (66.6%)	28 (73.7%)	
Unknown	1 (1.6%)	1 (3.4%)	0	
**Lesions number**				
1	21 (30.9%)	10 (33.3%)	11 (29%)	0.79
≥ 2	45 (66.2%)	19 (66.3%)	26 (68.4%)	
Unknown	2 (2.9%)	1 (0.4%)	1 (2.6%)	

### Comprehensive Profiling and Mutational Signatures in the Xuanwei Cohort

**Figure 3:**
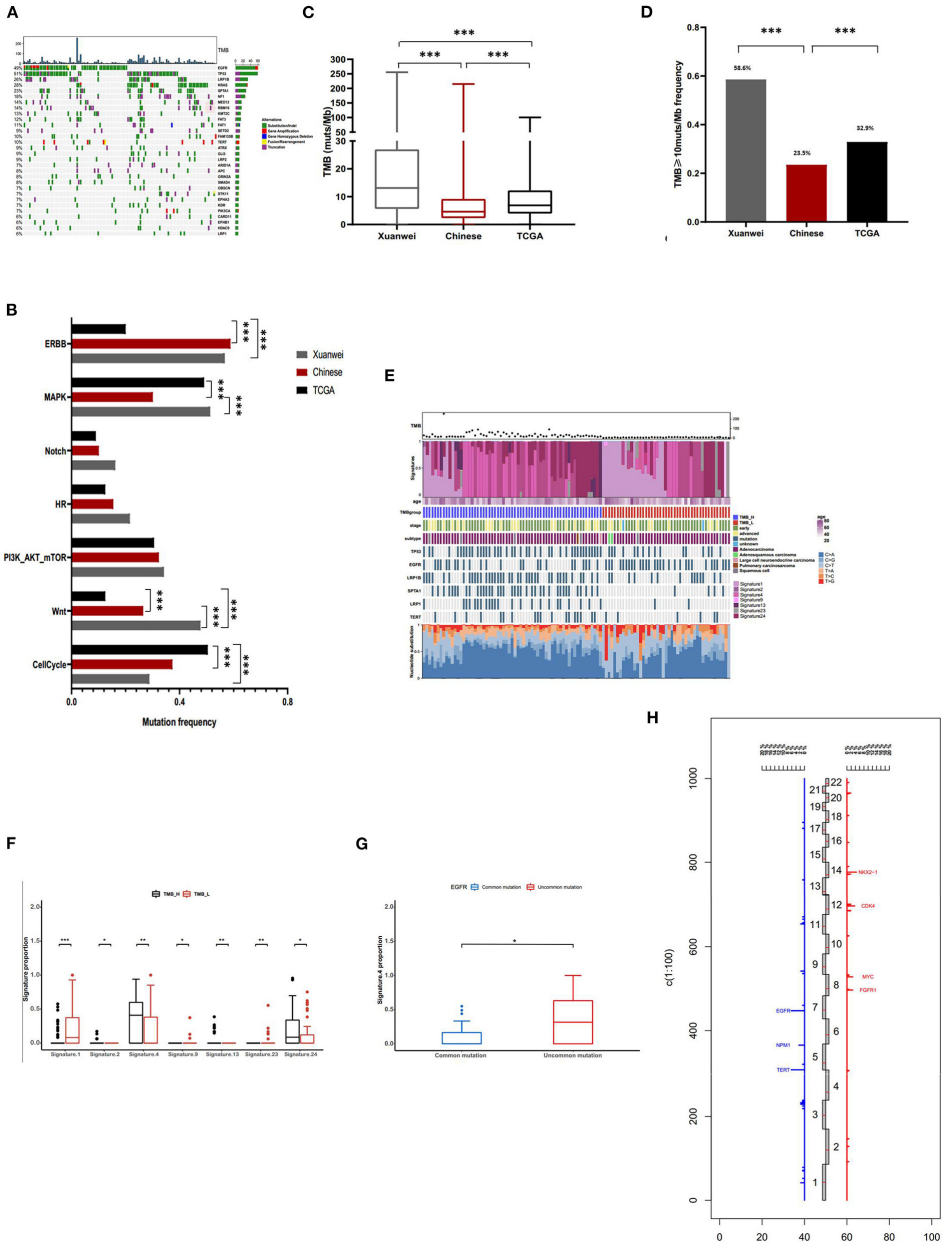
Comprehensive profiling of and somatic mutation signatures in patients from Xuanwei with NSCLC. **(A)** Comprehensive profiling of 111 patients from the Xuanwei cohort. **(B)** Comparison of the detection rate of gene mutations in pathways in the three cohorts. **(C)** Comparison of the proportion of the TMB value and **(D)** patients with TMB-H. **(E)** Somatic mutation signatures of the Xuanwei cohort. **(F,G)** Correlation between TMB value/*EGFR* mutation type and mutation signature. **(H)** Frequency of copy number variations of all chromosomal changes. Numbers 1–22 in the middle represent the human chromosome number. ***p < 0.001, **p < 0.01, and *p < 0.05.

The most commonly mutated genes in the Xuanwei NSCLC cohort were *TP53* (51%), *EGFR* (49%), *KRAS* (28%), *LRP1B* (26%), and *SPTA1* (23%) (Figure 3A). The mutation statuses of NSCLC-related pathways were analysed. Genes involved in these signalling pathways that were included in the 450-gene panel are listed in Supplementary Table 3. Gene mutations in the Wnt/MAPK/ERBB signalling pathways were the most common in patients from Xuanwei with NSCLC (Figure 3B). The Xuanwei cohort showed a higher median TMB than the reference Chinese cohort and TCGA cases (13.1, 4.6, and 6.9 muts/Mb, respectively; *p* < 0.001) (Figure 3C). TMB-H was detected in 58.6% of the Xuanwei cohort and 23.5% of the reference Chinese cohort (*p* < 0.001) (Figure 3D). TMB and mutational signatures reflect the process of mutation accumulation in cancer. To gain further insights into the mutational process of patients from Xuanwei, we characterised the mutation signatures via analysis of TMB-H and TMB-L tumours using the somatic mutation data. The profile of somatic mutations is shown in Figure 3E. We identified 3,596 single nucleotide variants and 58 insertions/deletions from 111 paired sequences of NSCLC in Xuanwei cohorts. The six subtypes of base substitutions (C > A, C > G, C > T, T > A, T > C, and T > G) were unevenly represented in single nucleotide variants. C > A was the most common substitution (1,685, 49.8%), followed by C > T (752, 22.2%) (Figure 3E).

We analysed the Spearman correlation coefficient between TMB and mutational signatures. Four predominant signatures (APOBEC, Smoking, Signature 13, and Signature 24) were observed in tumours with TMB-H, whereas Signature 1, Signature 9, and Signature 23 showed a correlation with TMB-L, suggesting the extensive accumulation of mutations due to smoking and exposure to aflatoxin. Although Signature 24 has been found in cancer samples from patients with known exposure to aflatoxin (supported by COSMIC data) (Figure 3F), further exploration of this association is necessary to confirm this hypothesis. Uncommon *EGFR* mutations showed high frequency about smoking-associated signature (Figure 3G).

Copy number variation analysis showed that the most frequently amplified genes were *EGFR* (6.3%), *TERT* (6.3%), *NKX2-1* (4.5%), *CDK4* (3.6%), *FGFR1* (2.7%), *MYC* (2.7%), *NMP1* (2.7%), and *SDHA* (2.7%) (Figure 3H). The association between gene mutations and TMB was analysed (Supplementary Figure 2A); among the common actionable mutations, *EGFR* mutation was significantly inversely correlated with TMB (6.9 vs. 17 muts/Mb, *p* < 0.001). Tumours with uncommon *EGFR* mutations showed a significantly higher TMB than those with common *EGFR* mutations (11.2 vs. 3.1 muts/Mb, *p* < 0.01) (Supplementary Figures 2A,B). *KRAS* mutation cases showed a higher median TMB than *KRAS* wild-type mutation cases (21.1 vs. 9.6 muts/Mb, *p* < 0.01). Tumours with *KRAS* G12C showed a lower TMB than those with non-*KRAS* G12C mutations (12 vs. 20.5 muts/Mb, *p* < 0.05) (Supplementary Figures 2A,B). *TP53, LRP1B, SPTA1, NF1*, and *KMT2C* mutations frequently occurred in patients with NSCLC and were significantly positively correlated with TMB (*p* < 0.001) (Supplementary Figure 2C).

### Antitumor Activity of EGFR-TKI in Patients From Xuanwei With Uncommon *EGFR* Mutations

Nine patients from Xuanwei with advanced lung adenocarcinoma bearing uncommon *EGFR* mutations, with a median age of 59 years, started EGFR-TKI treatment. Detailed mutation characteristics and the outcome of EGFR-TKI treatment are shown in Table 3. Nine patients included in the efficacy assessment had the sensitive uncommon G719X (9/9, 100%) and S768I (8/9, 89%) mutations. Before EGFR-TKI treatment, five patients showed metastasis in the lungs, two were diagnosed with brain metastasis, and one was diagnosed with bone metastasis. Eight patients received EGFR-TKI as first-line therapy, and one patient received osimertinib as third-line therapy. As per RECIST 1.1 guidelines, five patients achieved partial response or complete response, three showed stable disease, and the one that received osimertinib as third-line therapy showed progressive disease. Treatment-related adverse events included rash (2/9), pruritus (2/9), headache (2/9), stomatitis (2/9), and constipation (1/9). No grade 3 or more adverse events were identified. At data cut-off (February 1, 2020), three patients presented sustained disease control and remained on EGFR-TKI treatment, while the other six showed progressive disease. Four patients provided serial plasma to characterise the acquired resistance mechanisms: *EGFR* mutation loss was observed in two patients at the time of EGFR-TKI treatment. *MET* amplification emerged post-EGFR-TKI treatment in one patient; no resistance mechanisms were detected in the remaining patients via NGS. As blood analysis is not 100% sensitive for the detection of acquired mutations, other genetic alterations may be associated with resistance.

**Table 3:** Mutation characteristics and outcome of EGFR-TKI treatment.

**Patient**	**Age (years)**	**Sex**	**Stage**	***EGFR* mutation**	**Best response**	**EGFR-TKI**	**Therapy**	**Progression-free survival (months)**	**Resistance mechanism**
1	70	Male	IV	*EGFR* p.G719S/p.S768I/ p.N1107D	SD	Afatinib	First-line	16	Disappearance of EGFR
2	70	Female	IV	*EGFR* p.G719S/ p.S768I/Amplification	SD	Afatinib	First-line	7	Unknown
3	68	Female	IV	*EGFR* p.G719S/ p.S768I	PR	Afatinib	First-line	8	Disappearance of EGFR
4	54	Female	IIIB	*EGFR* p.G719S/ p.S768I	CR, ongoing treatment	Afatinib	First-line	12	
5	70	Female	IV	*EGFR* p.G719C/p.S768I	PR	Gefitinib	First-line	11	*EGFR* p.G719C/p.S768I
6	48	Male	IV	*EGFR* p.G719C/p.S768I/ Amplification	PR, ongoing treatment	Osimertinib	First-line	8	
7	49	Male	IV	*EGFR* p.G719C/p.K714E /p.V717L	SD, ongoing treatment	Gefitinib	First-line	2	
8	59	Male	IV	*EGFR* p.G719C/p.S768I	PR	Gefitinib	First-line	12	*MET* amplification, *PTEN* L325V
9	52	Male	IV	*EGFR* p.G719C/p.S768I	PD	Osimertinib	Third-line	1	Unknown

*PD, progressive disease; PR, partial response; SD, stable disease*.

## Discussion

High mortality and incidence rates of lung cancer have been documented in Xuanwei County. Our study revealed that the Xuanwei cohort had a greater cancer-related family history compared with the reference Chinese cohort, and most patients from Xuanwei showed a family history of lung cancer (96.7%). However, we did not detect any germline mutations in patients with family history. Patients with uncommon *EGFR* mutations were more likely to have a family history of cancer than those with common *EGFR* mutations (*p* = 0.05). A multicentre case-control study suggested that environmental tobacco smoke exposure could influence the *EGFR* mutation profile of lung cancer in never smokers and that exposure during adulthood might reduce the probability of *EGFR* mutation (17). Uncommon *EGFR* mutations and TMB-H were the predominant genomic features of patients with NSCLC from Xuanwei. Mutational signatures analysis demonstrated that uncommon *EGFR* mutations and TMB-H were correlated with smoking signature and we speculate that the environment (smoky coal) in Xuanwei may influence molecular features of patients from Xuanwei with NSCLC, which requires further analysis.

Patients with NSCLC harbouring *EGFR* mutations may benefit from EGFR-TKI therapy (18–20). The “uncommon” *EGFR* mutations account for 10–18% of all *EGFR* mutations, and NGS testing can broaden the spectrum of aberrations within the “uncommon group” in patients with NSCLC (21). Patients with *EGFR* mutations, including L858R, ex19del, and T790M, show a good response rate to first- or third-generation EGFR-TKIs, whereas those with uncommon *EGFR* mutations generally exhibit less benefit from targeted therapy (22). In our study, the NGS-based analysis of patients from Xuanwei with NSCLC revealed their comprehensive and unique profile of genomic alterations; uncommon *EGFR* mutations, mainly including G719X, S768I, and L861Q, were the predominant *EGFR* mutation types in the Xuanwei cohort, forming a distinctive subgroup of NSCLC globally. For patients with such non-classical *EGFR* mutations, previous studies illustrated that progression-free survival was significantly longer after afatinib treatment than that after first-generation EGFR-TKI (gefitinib/erlotinib) treatment (11.3 vs. 3.6 months, *p* = 0.03) (23). Osimertinib shows favourable activity with manageable toxicity in patients with metastatic or recurrent NSCLC harbouring uncommon *EGFR* mutations, achieving a median progression-free survival of 8.2 months (95% CI, 5.9 to 10.5 months) (24). According to our study, patients from Xuanwei with NSCLC harbouring G719X/S768I co-mutations may benefit from first-, second-, and third-generation EGFR-TKI treatment. The efficacy of these EGFR-TKIs in advanced patients from Xuanwei with uncommon *EGFR* mutations requires further analysis. Adjuvant therapy with gefitinib led to significantly longer disease-free survival in patients with completely resected stage II–IIIA NSCLC with *EGFR* mutations (exon 19del and exon 21 L858R) than platinum-based chemotherapy (25). Whether patients from Xuanwei with uncommon *EGFR* mutations could benefit from adjuvant EGFR-TKI treatment is also worth further investigation.

Acquired resistance to EGFR-TKIs is a common event, and several mechanisms, including T790M, *MET* amplification, and *PTEN* down-regulation, have been reported for the common *EGFR* mutations exon 19del and L858R (26). However, mechanisms underlying EGFR-TKI resistance have not been investigated for uncommon *EGFR* mutations. Our study revealed that *MET* amplification and loss of *EGFR* mutation loss were related to EGFR-TKI resistance in patients harbouring *EGFR* G719X/S768I co-mutations. Nevertheless, further studies are required to confirm these mechanisms.

*KRAS* is a G-protein with intrinsic GTPase activity. *KRAS* mutations are associated with reduced responsiveness to EGFR-TKI therapy and poor survival (27). The *KRAS* mutation frequency in the Xuanwei cohort was significantly higher than that in the reference Chinese cohort. Furthermore, *KRAS* G12C accounted for 53.8% of *KRAS*-mutant patients in the Xuanwei cohort. AMG 510 is a novel, first-in-class, small molecule that specifically and irreversibly inhibits KRAS G12C by permanently locking it in an inactive GDP-bound state, and it demonstrated promising antitumor activity in patients with advanced NSCLC harbouring the *KRAS* G12C mutation (28). Patients from Xuanwei, who harbour the *KRAS* G12C mutation, may benefit from this inhibitor.

The TMB is an evolving biomarker for identifying eligible patients for checkpoint inhibitors in NSCLC (29, 30). Patients from Xuanwei with NSCLC showed a much higher median TMB than those in the reference Chinese and TCGA cohorts. Thus, patients from Xuanwei with NSCLC may benefit from immunotherapy. Furthermore, the TMB of patients with uncommon *EGFR* mutations was significantly higher than that of patients with common *EGFR* mutations in the Xuanwei cohort. Whether patients with uncommon *EGFR* mutations can benefit from immunotherapy requires further investigation.

This study comprehensively elucidated the molecular features of patients from Xuanwei with NSCLC via NGS. These patients appear to have a higher uncommon *EGFR* mutation ratio and a higher TMB value than reference Chinese patients and TCGA cohorts, and patients with uncommon *EGFR* mutations seem to show a good response to EGFR-TKI therapy. Studies with larger cohorts are needed to validate these observations, and further clinical research is warranted to provide insights into how comprehensive genomic profiling can guide treatment decisions for patients from Xuanwei with NSCLC.

## Data Availability Statement

The datasets presented in this study can be found in online repositories. This data can be found here: https://db.cngb.org/cnsa/review/show/CNP0001608_20210319_9919b624/.

## Ethics Statement

The studies involving human participants were reviewed and approved by First Hospital of Kunming Medical University. The patients/participants provided their written informed consent to participate in this study.

## Author Contributions

YS, TS, and GG contributed to conception and design of the study. HL, QG, JL, and XY provided study or patients material. GL and YL collected and/or assembled data. GL, YL, and YS performed the statistical analysis and interpreted data. YS, TS, GG, and YL wrote the manuscript. All authors contributed to manuscript revision, read, and approved the submitted version.

## Conflict of Interest

The authors declare that the research was conducted in the absence of any commercial or financial relationships that could be construed as a potential conflict of interest.

## Abbreviations

APOBEC: apolipoprotein B mRNA editing enzyme catalytic polypeptide-like
COSMIC: Catalogue of Somatic Mutations in Cancer
Mb: megabase
NGS: next-generation sequencing
NSCLC: non-small cell lung cancer
RECIST: Response Criteria in Solid Tumours
TCGA: The Cancer Genome Atlas
TKI: tyrosine kinase inhibitor
TMB: tumour mutation burden
TMB-H: high TMB
TMB-L: low TMB
CT: computed tomography.
