# Supplementary figures and images for: Next-Generation Sequencing Reveals High Uncommon EGFR Mutations and Tumour Mutation Burden in a Subgroup of Lung Cancer Patients
Source: Front Oncol. 2021 Apr 6;11:621422. doi: 10.3389/fonc.2021.621422 (PMC8056083; doi:10.3389/fonc.2021.621422)

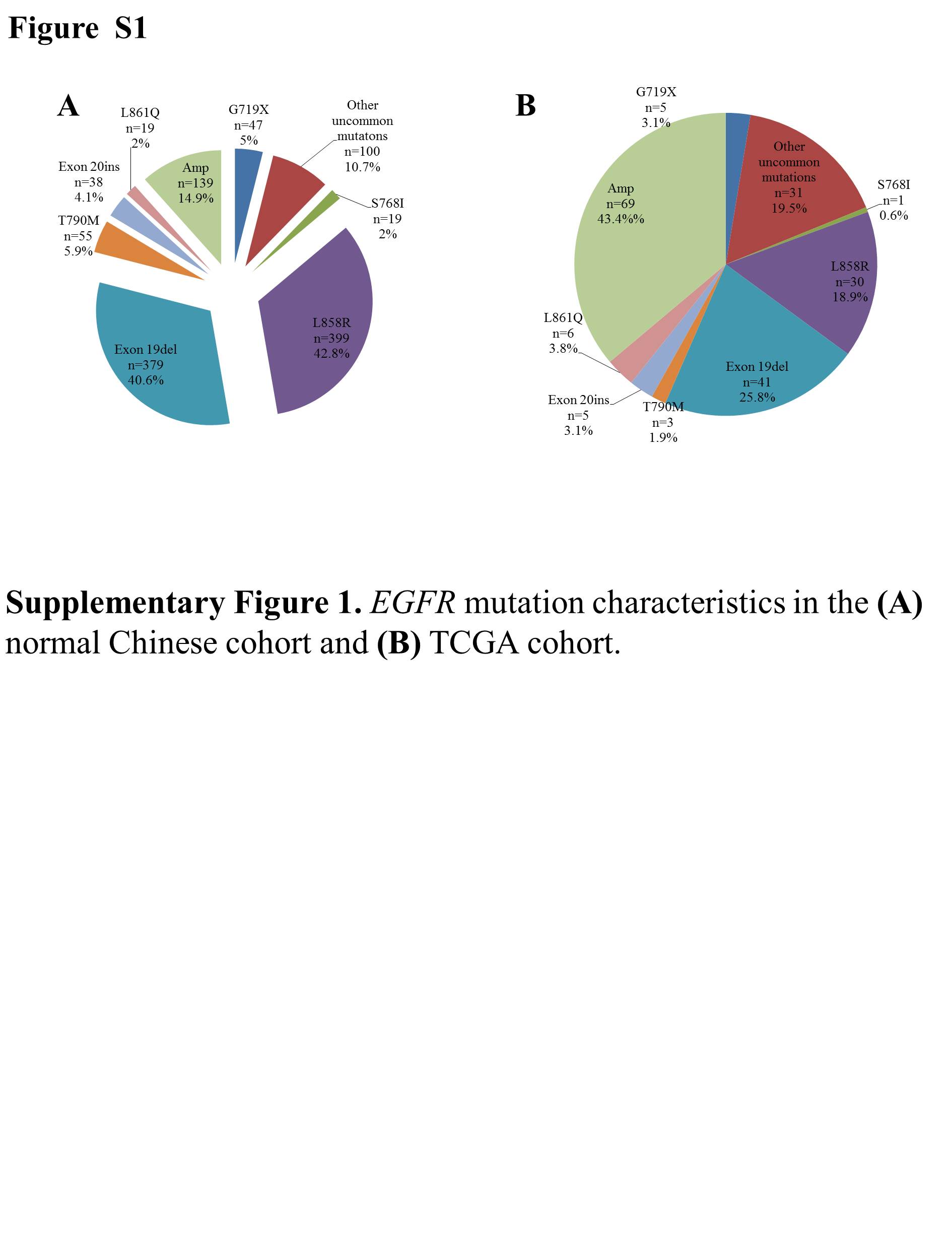

Supplement: Supplementary file 4 [file Image_1.JPEG]

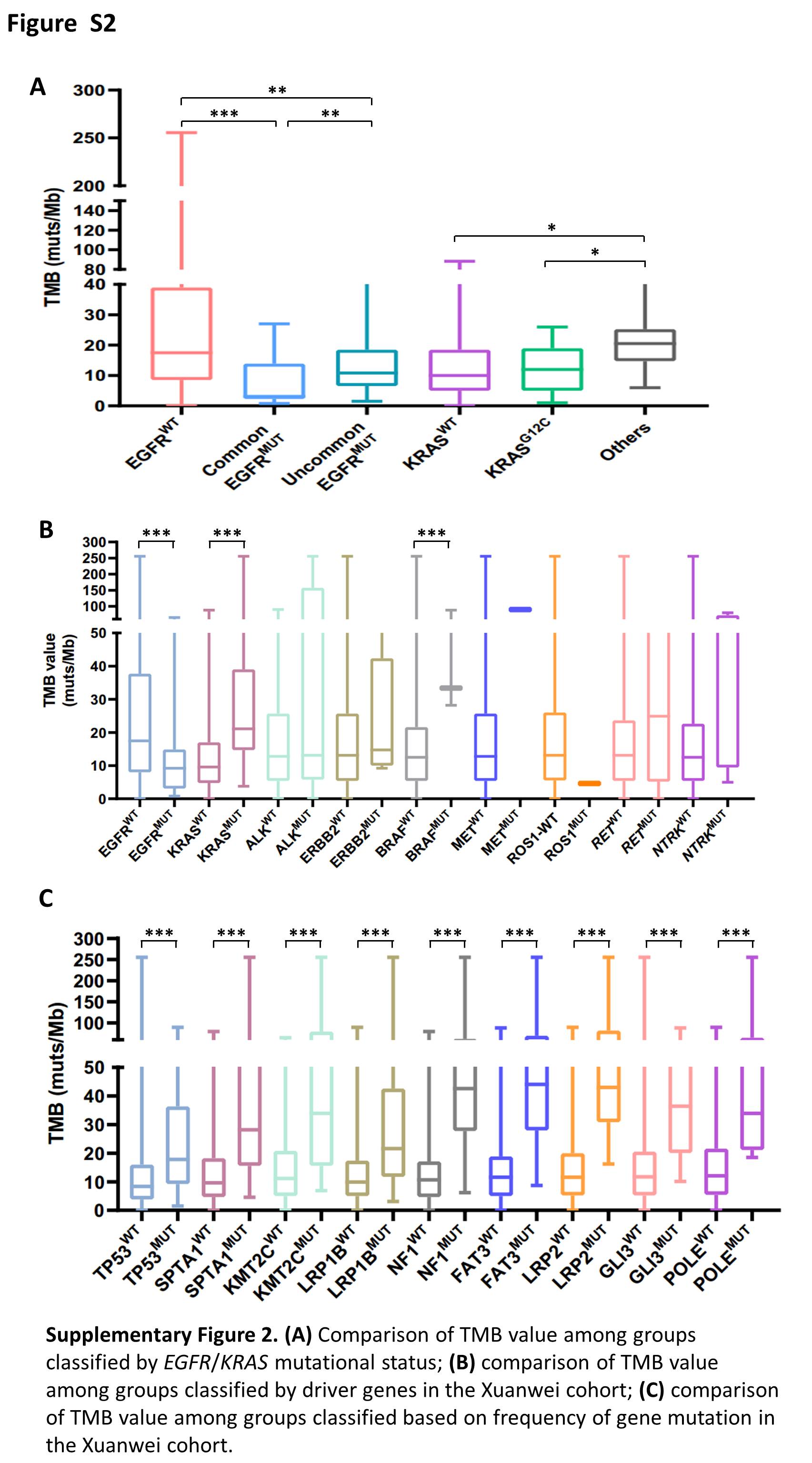

Supplement: Supplementary file 5 [file Image_2.JPEG]
